# Labour and Health

**Published:** 1894-09-15

**Authors:** 


### Modern Sociology.

### LABOUR AND HEALTH.—I.

It is now nearly a hundred years since the conditions of labour began to be recognised as a proper subject for legislation. Labour laws of the drastic kind, which consisted in flogging the idle at the cart-tail and sternly limiting the wage of the industrious, were the only interference between employer and employed deemed proper and safe by our ancestors, and it is but fair to remember that laws enforcing in factories the commonest elements of decency and humanity were in the first instance resisted quite as strenuously by the men as by the masters. Interference with the liberty of a British subject to over-work, starve, stifle, poison, burn, or cut to pieces himself or anyone else in the way of business was, at the outset of the matter, considered intolerable, and the earliest reforms were carried in the teeth of public opinion and under the gravest apprehensions of national disaster. The whole struggle, beginning in real earnest with the passing of the Factory Health and Morals Act, 1802, is concisely set forth by Dr. Cooke-Taylor in the new volume of "Social Questions of the Day."[1] In this small compass a fairly complete survey is given of the progress of the social revolution or rather evolution now in process of accomplishment in our vast manufacturing population, with some pertinent suggestions as to the lines of its further advance.

From first to last the State intervention between master and man has taken an ethical and sanitary, never an economic direction. The right of contract and the rate of wages have, with the single exception of the abolition of truck payment, never been even indirectly approached. The real work of the State during the present century has been slowly to make good its claim to a population of healthy men and women, and this claim, long tacitly conceded, has led to developments never dreamed of in the past and capable of almost infinite expansion in the future. "Impossible to make people healthy and virtuous by Acts of Parliament" was and still is the cry. Granted. But quite possible to provide that they shall not be compelled by the very conditions of their existence to be diseased and vicious. It was accordingly to preventive measures that the early reformers exclusively confined their efforts.

The principal stronghold of disease in the old factory system was admittedly the abuse of child labour, and round this subject, appealing more forcibly than any other could do to public sympathy, the brunt of the battle raged. It is needless to recall the mass of evidence accumulated under Sadler's Committee, which ledto the passing of the Factory Act of 1831. As late as the year 1864 children of two and two and a-half were found employed in the lace industry, and in the brick fields boys and girls of six and even four years of age were kept at work from four in the morning till nine at night. The very gravity of the abuse worked its own redress. Not the smallest argument in favour of State interference with the Government of the day was the glaring deficiency of suitable recruits for the army shown in the great manufacturing centres. Nature, to use Charles Kingsley's words, "killed and killed and killed" until she made her voice heard, and the public awoke to the perception that not the mere well-being but the actual existence of the manufacturing population was at stake. All subsequent reforms developed naturally from the position which the State was at last driven to assume of protector to the coming race; so truly might it be said in the matter of these labour laws, "And a little child shall lead them."

The first law imposing any real restriction on child labour (omitting the Factory Act of 1802, which dealt only with apprentices) was that introduced by Sir Robert Peel in 1815, and finally passed in 1819, providing that no child should be employed in cotton spinning under nine years of age, and no persons under sixteen for more than twelve hours a day, deducting one and a-half hours for meals. It has taken over seventy years of unceasing effort to give full effect to the principle inaugurated by this measure. To extend its benefits to children in every form of employment, to provide for their education, to lengthen their non-working years to eleven, and by the creation of the class of "young persons" to keep the growing citizen under supervision till the age of eighteen; to restrict the labour hours of women as mothers *in posse*, and prohibit their employment for four weeks after child-birth; to provide against injury to health from over-crowding, dirt, poisoned air, or dangerous machinery, and finally to ensure by rigid inspection the carrying out of the law; these have been some of the inevitable additions to the first subversive measure which pronounced the right of the State to a healthy rising generation.

Meantime, the male labourer of full years has been let sternly alone, enjoying only a certain measure of protection from the presence of women or children in his workshop. It is not wonderful that, awaking to a new sense of the beauty and dignity of life, he is beginning to ask why the laws, which ensure him a healthy childhood and youth, should leave him as he enters manhood to an unequal struggle with relentless competition. And it may be reckoned as the highest achievement of the State that the worker has learned from the experience of these remedial measures to centre his hope for a happier future, not on revolutionary upheaval, but on a wise readjustment of social law.

[1] "The Factory System." R. W. Cooke-Taylor, F.S.S. (Methuen and Co.)

